# Complement factor H contributes to mortality in humans and mice with bacterial meningitis

**DOI:** 10.1186/s12974-019-1675-1

**Published:** 2019-12-28

**Authors:** E. Soemirien Kasanmoentalib, Mercedes Valls Serón, Joo Yeon Engelen-Lee, Michael W. Tanck, Richard B. Pouw, Gerard van Mierlo, Diana Wouters, Matthew C. Pickering, Arie van der Ende, Taco W. Kuijpers, Matthijs C. Brouwer, Diederik van de Beek

**Affiliations:** 1 Department of Neurology, Amsterdam UMC, University of Amsterdam, Amsterdam Neuroscience, Amsterdam, the Netherlands; 2 Department of Clinical Epidemiology, Biostatistics and Bioinformatics, Amsterdam UMC, University of Amsterdam, Amsterdam, the Netherlands; 3 Department of Immunopathology, Sanquin Research and Landsteiner Laboratory of the Academic Medical Center, University of Amsterdam, Amsterdam, the Netherlands; 4 Department of Pediatric Hematology, Immunology and Infectious Diseases, Emma Children’s Hospital, Amsterdam UMC, Amsterdam, the Netherlands; 5 Centre for Inflammatory Disease, Division of Immunology and Inflammation, Department of Medicine, Imperial College London, London, UK; 6 Department of Medical Microbiology and The Netherlands Reference Laboratory for Bacterial Meningitis, Amsterdam UMC, University of Amsterdam, Amsterdam, the Netherlands; 7 Department of Blood Cell Research, Sanquin Research and Landsteiner Laboratory of the Academic Medical Center, University of Amsterdam, Amsterdam, the Netherlands

**Keywords:** Bacterial meningitis, Pneumococcal meningitis, Complement system, Complement factor H, Anti-inflammatory therapy, Animal models

## Abstract

**Background:**

The complement system is a vital component of the inflammatory response occurring during bacterial meningitis. Blocking the complement system was shown to improve the outcome of experimental pneumococcal meningitis. Complement factor H (FH) is a complement regulatory protein inhibiting alternative pathway activation but is also exploited by the pneumococcus to prevent complement activation on its surface conferring serum resistance.

**Methods:**

In a nationwide prospective cohort study of 1009 episodes with community-acquired bacterial meningitis, we analyzed whether genetic variations in *CFH* influenced FH cerebrospinal fluid levels and/or disease severity. Subsequently, we analyzed the role of FH in our pneumococcal meningitis mouse model using FH knock-out (*Cfh*^−/−^) mice and wild-type (wt) mice. Finally, we tested whether adjuvant treatment with human FH (hFH) improved outcome in a randomized investigator blinded trial in a pneumococcal meningitis mouse model.

**Results:**

We found the major allele (G) of single nucleotide polymorphism in *CFH* (rs6677604) to be associated with low FH cerebrospinal fluid concentration and increased mortality. In patients and mice with bacterial meningitis, FH concentrations were elevated during disease and *Cfh*^−*/*−^ mice with pneumococcal meningitis had increased mortality compared to wild-type mice due to C3 depletion. Adjuvant treatment of wild-type mice with purified human FH led to complement inhibition but also increased bacterial outgrowth which resulted in similar disease outcomes.

**Conclusion:**

Low FH levels contribute to mortality in pneumococcal meningitis but adjuvant treatment with FH at a clinically relevant time point is not beneficial.

## Background

Despite the introduction of effective vaccines and antibiotic treatment pneumococcal meningitis is the most common and severe form of bacterial meningitis and is associated with mortality rates of 18 to 37% and neurological sequelae in about 50% of survivors [1–6]. Due to serotype replacement reducing vaccine effectiveness and the growing emergence of antibiotic resistance new treatment strategies are needed [7–9]. Experimental bacterial meningitis animal models have shown that outcome is related to the severity of inflammation in the subarachnoid space and that the outcome can be improved by modulation of this inflammatory response [10–12]. The introduction of dexamethasone, an anti-inflammatory drug, has improved outcomes in patients in high-income countries although the burden of disease remains substantial [13–16]. Other adjunctive therapies are needed to further improve the prognosis of pneumococcal meningitis patients [17].

The complement system was shown to play an important role in the inflammatory response during pneumococcal meningitis [10, 18–21]. Several experimental studies have evaluated the blocking of the complement system using antibodies to improve the outcome of pneumococcal meningitis [22–24]. Inhibition of complement component 5 (C5), blocking the common terminal pathway of the complement system was shown to reduce inflammation and improve the outcome of experimental pneumococcal meningitis [22, 23]. However, treatment with C5 antibodies blocks the terminal complement pathway impairing the killing of *Neisseria meningitidis*, the second most common cause of bacterial meningitis [25]. Moreover, intervening in the complement system upstream in one of the three activation pathways could be more beneficial as it also reduces early anaphylatoxin formation. The classical, lectin, and alternative pathway of complement all lead to the formation of the opsonin C3b, which also induces the formation of C3 and C5 convertase when binding to other complement components. The alternative pathway is activated by spontaneous hydrolysis of C3, resulting in the formation of a solvent-based C3 convertase followed by the deposition of C3b on cell surfaces. Once C3b has been formed, by one of the three pathways, the alternative pathway amplification loop can rapidly increase C3b production [26, 27].

Complement factor H (FH) is a soluble plasma protein that plays a critical role in the inhibition of alternative pathway activity on host cells [28, 29]. FH regulates the alternative pathway by preventing the formation of the alternative pathway C3 convertase by binding to C3b on host cells, by promoting the dissociation of C3 convertase and acts as a co-factor in the factor I mediated inactivation of C3b [28, 30]. Patients with complete FH deficiency have uncontrolled alternative pathway activation and secondary C3 depletion associated with bacterial infections, atypical hemolytic uremic syndrome, and membranoproliferative glomerulonephritis [28, 31].

Several studies showed genetic variation in the FH gene, *CFH*, influences the risk of infectious diseases and influenced bacterial killing in vitro [32–36]. FH blood levels were furthermore shown to influence bacterial outgrowth of *Streptococcus pneumoniae* in vitro and in vivo [37]. In a model of autoimmune encephalomyelitis, FH treatment was shown to decrease inflammation in the central nervous system and thereby disease severity [38]. Modulating the alternative pathway by targeting FH may therefore be an effective adjuvant treatment to reduce the inflammatory response and thereby improve outcomes in pneumococcal meningitis.

We evaluated the role of FH in pneumococcal meningitis: first, we analyzed whether genetic variations in *CFH* in bacterial meningitis patients influenced disease severity, then measured FH in the cerebrospinal fluid (CSF) and performed immunohistochemistry staining for FH in brains of bacterial meningitis patients to determine if and where FH is expressed during meningitis. Subsequently, we analyzed the role of FH in our pneumococcal meningitis mouse model using FH knock-out (*Cfh*^−/−^) mice and wild-type (wt) mice. Finally, we tested whether adjuvant treatment with human FH (hFH) improved outcome in a randomized investigator blinded trial in a pneumococcal meningitis mouse model.

## Methods

### Patient cohort

The MeninGene study is a nationwide prospective cohort study of adults with community-acquired bacterial meningitis. The cohort and methods have been described previously [1]. Clinical data was collected using online case record forms and included patients characteristics, clinical and laboratory parameters, treatment, and outcome. The outcome was graded at discharge according to the Glasgow Outcome Scale (GOS), a well-validated instrument [39]. A score of 1 indicates death; a score of 2 indicates a vegetative state; a score of 3 indicates severe disability; a score of 4 indicates moderate disability; and a score of 5 indicates mild or no disability. A favorable outcome was defined as a score of 5 and unfavorable outcome as a score of 1 to 4. The study was approved by the medical ethical committee of the Academic Medical Centre, Amsterdam, the Netherlands.

#### Genetics

Blood from patients was collected in sodium/EDTA for DNA extraction. DNA was isolated with the Gentra Puregene Isolation Kit (Qiagen, Hilden, Germany) and quality control procedures were performed to determine the yield of isolation. To determine whether genetic variance in *CFH* influences the outcome of bacterial meningitis we performed a genetic association study for four common functional single-nucleotide polymorphisms (SNP) in *CFH* (rs6677604, rs1065489, rs3753394, rs800292).

#### Cerebrospinal fluid

Residual CSF from the diagnostic lumbar puncture was collected from bacterial meningitis patients. CSF samples from 18 patients with benign thunderclap headache in whom a lumbar puncture was done to exclude a subarachnoid hemorrhage and had normal CSF examination were used as controls. The CSF was centrifuged and the supernatant was stored at − 80 °C until analysis. FH, C3a, C5a, and C5b-9 levels were determined by ELISA according to manufacturer’s instructions (Microvue Quidel, San Diego, CA, USA). Part of the CSF data have been published previously [20].

#### Brain pathology

Brain tissue from a pneumococcal meningitis patient and a control patient with myocardial infarction without history of the neurological disease was available through the AMC neuropathology biobank to evaluate whether FH could be visualized during pneumococcal meningitis [40]. Paraffin-embedded brain tissue was deparaffinized and endogenous peroxidases were blocked by incubation with 0.3% hydrogen peroxide in methanol (EMSURE®). Sections were incubated with mouse anti-human FH antibodies (clone anti-FH.16, binds domain 16/17, Sanquin Research, Amsterdam, the Netherlands) in normal antibody diluent (BrightVision, ImmunoLogic). Bound primary antibody was blocked and detected using poly streptavidin horseradish peroxidase goat anti-mouse/rabbit/rat IgG and diaminobenzidine which yields a brown reaction product. Counterstaining was performed using hematoxylin.

### Pneumococcal meningitis mouse model

To determine the role of FH during pneumococcal meningitis we used our well-validated pneumococcal mouse model [41]. C57BL/6NCrl mice (Charles River Laboratory), aged 8–12 weeks old, were injected in the cisterna magna with 1μl of 10^7^ CFU/ml *S. pneumoniae* serotype 3 (ATCC 6303; American Type Culture Collection, Rockville, MD, USA) or saline under isoflurane anesthesia. All animals were clinically examined before and directly following inoculation and at regular intervals. The scoring list includes weight loss, activity, time to return to an upright position, state of fur, posture, eye discharge or protrusion, respiration rate, irregular/labored breathing, neurological deficits, and epilepsy. A score of 15 or more was defined as a humane endpoint, other humane endpoints were > 25% weight loss, ≥ 2 seizures per 15 min, status epilepticus, and hemiparalysis. Mice were euthanized when reaching a humane endpoint or at predefined time points by intraperitoneal injection of ketamine (190 mg/kg) and dexmedetomidine (0.3 mg/kg). Blood was collected by cardial puncture and citrated in a 1:4 citrate to blood ratio, CSF was collected by puncture of the cisterna magna. Subsequently, mice were perfused with sterile phosphate-buffered saline (PBS) and the left hemisphere, spleen and lung were harvested and processed as described before [41]. The right cerebral hemisphere was fixed in 10% buffered formalin and embedded in paraffin for histopathology. Bacterial titres were determined by plating serial ten-fold dilutions of blood, CSF, brain, spleen, and lung homogenates on sheep-blood agar plates and incubating for 16 h at 37 °C. Plasma, CSF, and lysed supernatant were stored at − 80 °C until assayed. Animal experiments were approved by the Institutional Animal Care and Use Committee of the Academic Medical Center Amsterdam.

#### FH expression experiments

Pneumococcal meningitis was induced at *t* = 0 and mice were sacrificed at 6 (*n* = 5), 24 (*n* = 5), and 48 h (*n* = 5) after infection. Mice in the 48 h group were treated intraperitoneally with ceftriaxone (100 mg/kg) at 20 h after infection. Mice inoculated with sterile saline were sacrificed at 24 h (*n* = 5) and served as control.

#### FH deficiency experiments

First, a survival study was performed where wild-type (wt) mice and FH deficient mice (*Cfh*^*−/−*^, *n* = 12 per group) with a C57BL/6 background were infected and observed for 50 h. *Cfh*^*−/−*^ mice were a kind gift of Prof. M.C. Pickering (Imperial College London, UK) and are described elsewhere [42]. In a time-point experiment, mice were infected and euthanized at 5 (*n* = 10 per group) and 20 h (*n* = 11 per group) after infection.

#### Purification of plasma-derived human FH

Human FH was purified from a combined citrated plasma pool of four healthy donors. Fresh frozen plasma was thawed at 4 °C and separated from cryoprecipitates by filtration using KS700 and KS50 filters (Pall, 0.45 μm cut-off). Obtained filtrate, diluted in 20 mM Tris, pH 8.0, was loaded onto a DEAE sepharose FF (GE Healthcare) column (475 mL) and eluted by a step-wise gradient (7.5%, 12.5%, and 100% v/v) of 1 M NaCl in 20 mM Tris, pH 8.0. Collected fractions containing FH were pooled, diluted with 0.1 M NaAc buffer, pH 5.7, loaded onto a HiPrep CM FF 16/10 20 mL column (GE Healthcare) and eluted with a linear gradient of 1 M NaCl in 0.1 M NaAc, pH 5.7 to 25% (v/v), followed by stepwise elution at 50% (v/v) and 100% (v/v) 1 M NaCl in 0.1 M NaAc, pH 5.7. Collected fractions containing FH were pooled, diluted with 20 mM Tris, pH 7.4, loaded onto a HiPrep MonoQ XL 16/10 20 mL column (GE Healthcare) to remove endotoxins and concentrate FH, and eluted with 0.5 M NaCl in 20 mM Tris, pH 7.4. Collected fractions containing FH were further concentrated using a 10 kDa cut-off Amicon filter (Millipore) followed by a Sephacryl S200 HR (Ge Healthcare) column (2 L) using PBS as running buffer. The presence of FH in collected fractions was confirmed by hFH ELISA and SDS-PAGE. All steps were performed at 4–8 °C and samples were kept at 4 °C between runs. The purity of obtained hFH was assessed by SDS-PAGE (> 97% pure). The activity of hFH was confirmed by co-factor activity assay and binding to C3b, assessed by SPR, as previous described (Pechtl et al, Schmidt et al.). Purified hFH was stored in PBS at − 80 °C until use.

#### Adjuvant treatment with human FH

It has been shown that hFH is capable to inhibit mouse complement activity [38, 43, 44]. To evaluate the effect of adjuvant treatment with hFH in pneumococcal meningitis mice were treated intraperitoneally 16 h after infection with plasma-derived hFH (1 mg) or PBS. At the same time, mice were treated with intraperitoneal ceftriaxone (100 mg/kg) which was repeated daily (16, 40, and 64 h). In a survival experiment, mice were observed during 72 h (*n* = 12 per group). In a time point, experiment mice were euthanized at 24 (*n* = 11 per group) and 48 h (*n* = 11 per group). In a second survival experiment, mice were treated from 16 h with daily ceftriaxone and at 18 h with hFH (1 mg) or PBS (*n* = 12 per group). All mice received an identical total amount of fluids. Mice were randomly assigned to treatment groups using a computer-generated random number list (Microsoft Excel 2010), and all researchers were blinded for the treatment group. The randomization code was broken after the last experiment was finished.

#### Protein expression

IL-1β, IL-6, IL-10, KC, and MIP-2 levels were determined in mouse brain homogenates with ELISA (R&D Systems, MN, USA). Albumin concentrations in brain homogenates were determined with ELISA (ALPCO Diagnostics, Salem, USA). ELISA was used to measure FH in mouse brain homogenates (only detects FH and no FH-related proteins; Quidel, San Diego, USA) and C3 (MyBiosource) and C5b-9 (Wuhan USCN Business Co., Houston, USA) in mouse plasma and brain homogenates. Human FH was detected in mouse plasma and brain homogenates by ELISA, methods described elsewhere [45].

#### Murine brain pathology

The paraffin-embedded right hemisphere was cut in coronal sections of 5 μm. Human FH was detected in the brains of hFH-treated mice by staining with anti-FH.16 as described above [45].

#### Statistics

Continuous variables were compared using the Mann-Whitney *U* test and the Kruskal-Wallis test when comparing more than two groups. Dichotomous variables were compared using the Pearson chi-squared test. Survival was analyzed using a log-rank test. Clinical scores were compared using exponential regression: *score* =  − 1 × e^b ∗ Time (h)^ with a random slope and assuming an autoregressive correlation structure of order 1. For all analyses, a *P* value < 0.05 was regarded as significant.

## Results

### Nationwide prospective cohort study of community-acquired bacterial meningitis

Between January 2006 and October 2011, 1009 episodes of community-acquired bacterial meningitis were included in our nationwide cohort study. The median age was 60 years (interquartile range 45–69) and 509 (49%) were female (Table 1). Predisposing conditions for meningitis were identified in 57% of patients, and 26% were immunocompromised. *S. pneumoniae* was the causative pathogen in 727 patients (72%), *N. meningitidis* in 111 (11%) and other bacteria in 171 patients (17%). A total of 182 (18%) died and 391 patients (39%) had an unfavorable outcome defined as a score of 1 to 4 on the GOS [39].

**Table 1:** Baseline characteristics of 1009 episodes of community-acquired bacterial meningitis^a^

Clinical characteristics	n/N (%)
Age (years)	60 (45–69)
Male	509 (49%)
Predisposing conditions	580/1009 (57%)
Otitis or sinusitis	329/1007 (24%)
Pneumonia	90/975 (9%)
Immunocompromised state b)	258/1009 (26%)
Symptoms and signs at admission	
Symptoms < 24 h	452/973 (46%)
Headache	742/891 (83%)
Neck stiffness	722/956 (76%)
Temp > 38 °C	757/1002 (76%)
Score on Glasgow coma scale c)	11 (9–14)
< 8 indicating coma	135/1008 (13%)
CSF values d)	
White blood cell count (/μl)	2497 (561–7561)
Protein (g/l)	3.82 (2.30–5.99)
CSF/blood glucose ratio	0.03 (0.00–0.25)
Causative pathogen	
*S. pneumoniae*	727 (72%)
*N. meningitidis*	111 (11%)
Other	171 (17%)
Score on Glasgow outcome scale	
1—death	182 (18%)
2—vegetative state	1 (0.1%)
3—severe disability	48 (5%)
4—moderate disability	160 (16%)
5—good recovery	618 (61%)

^a^Data are number/number evaluated (%) or median (interquartile range).

^b^Immunocompromise was defined by the use of immunosuppressive drugs, a history of splenectomy, or the presence of diabetes mellitus, alcoholism, as well as patients infected with the human immunodeficiency virus (HIV)

^c^Score on the Glasgow Coma Scale Score was evaluated in 1008 patients

^d^CSF white blood cell count was determined in 970 patients, CSF proteins levels in 963 patients and the CSF/blood glucose ratio in 946 patients

### Genetic variation in *CFH* influences outcome of bacterial meningitis

**Fig. 1:**
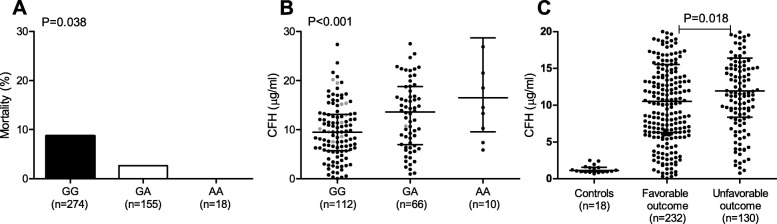
Association of SNP rs6677604 with mortality and cerebrospinal fluid FH concentration in patients with bacterial meningitis. In immunocompetent bacterial meningitis patients of Caucasian descent, the rs6677604 major allele G (minor allele frequency A EUR 0.187) was associated with an increased mortality under an additive model (**a**). *P* value was determined with the Pearson chi-square test. The rs6677604 major allele G was also associated with lower FH CSF levels under an additive model (**b**). *P* value was determined with the Kruskal-Wallis test. In bacterial meningitis patients the CSF FH concentration was significant higher compared to controls (**c**). Bacterial meningitis patients with an unfavorable outcome had slightly increased FH levels compared to patients with a favorable outcome. After correction for CSF total protein CSF FH levels were similar in bacterial meningitis patients with a unfavourable and a favourable outcome. *P* value was determined with the Mann-Whitney *U* test. Each dot represents an individual patient, gray dots represent deceased patients, lines represent median values and error bars are interquartile ranges

To determine whether genetic variance in *CFH* influences outcome of bacterial meningitis, we performed a genetic association study of four common functional single nucleotide polymorphisms (SNP) in *CFH* (rs6677604, rs1065489, rs3753394, rs800292). DNA was available for 664 of the 1009 bacterial meningitis episodes (66%). Mortality was significantly higher in patients with no DNA available compared to those with DNA available (40% vs. 7%, *P* < 0.001; Additional file 1: Table S1). Genotyping was successful in 97% of patients. In immunocompetent bacterial meningitis patients of Caucasian ethnicity (456 of 664) the major allele G of rs6677604 (minor allele frequency A, EUR 0.187) was associated with an increased mortality compared to minor allele A using an additive model (Pearson chi-square *P* = 0.038; Fig. 1a). The rs3753394 CT/TT genotypes (minor allele frequency T EUR 0.267) were associated with an unfavorable outcome compared to the CC genotype using a dominant model (Pearson chi-square *P* = 0.047, OR 1.53 95%CI 1.00–2.34), but there was no association with mortality.

### Cerebrospinal fluid FH levels are increased during bacterial meningitis

To evaluate FH levels in the central nervous system during bacterial meningitis we measured FH levels in left-over CSF samples from the diagnostic lumbar puncture using ELISA. CSF samples from patients with benign thunderclap headache in whom a lumbar puncture was done to exclude a subarachnoid hemorrhage and had normal CSF examination were used as controls. CSF was available for 362 of the 1009 bacterial meningitis episodes (36%). Baseline characteristics were similar among patients with and without CSF available. Bacterial meningitis patients had increased FH levels compared to controls (median 11.27 [IQR 6.62–15.86] vs. 1.12 [IQR 0.93–1.55] μg/ml, *P* < 0.001; Fig. 1cs). All other measured complement factors were also increased in CSF of bacterial meningitis patients compared to controls (C3a, C5a, and C5b-9 *P* < 0.001) [20]. Patients with an unfavorable outcome had slightly higher CSF FH levels compared to those with an favorable outcome (median 11.92 [IQR 8.36–16.40] vs. 10.51 [IQR 6.28–15.54] μg/ml, *P* = 0.018). After correction for CSF total protein CSF FH levels were similar in bacterial meningitis patients with unfavorable and a favorable outcome. FH concentration was not significantly different between deceased patients compared to survivors (median 12.12 [IQR 7.08–18.09] vs. 11.06 [IQR 6.59–15.76] μg/ml, *P* = 0.28). In patients with rs6677604 GG genotype (associated with higher mortality), FH CSF levels were significantly lower compared to the GA and AA genotypes using an additive model (median 9.50 [IQR 5.72–13.13] vs. median 13.62 [IQR 6.94–18.78] and median 16.52 [IQR 9.55–28.69] μg/ml; Kruskal-Wallis test *P* < 0.001, Fig. 1b).

### Immunohistochemical staining of FH in brain of a pneumococcal meningitis patient

**Fig. 2:**
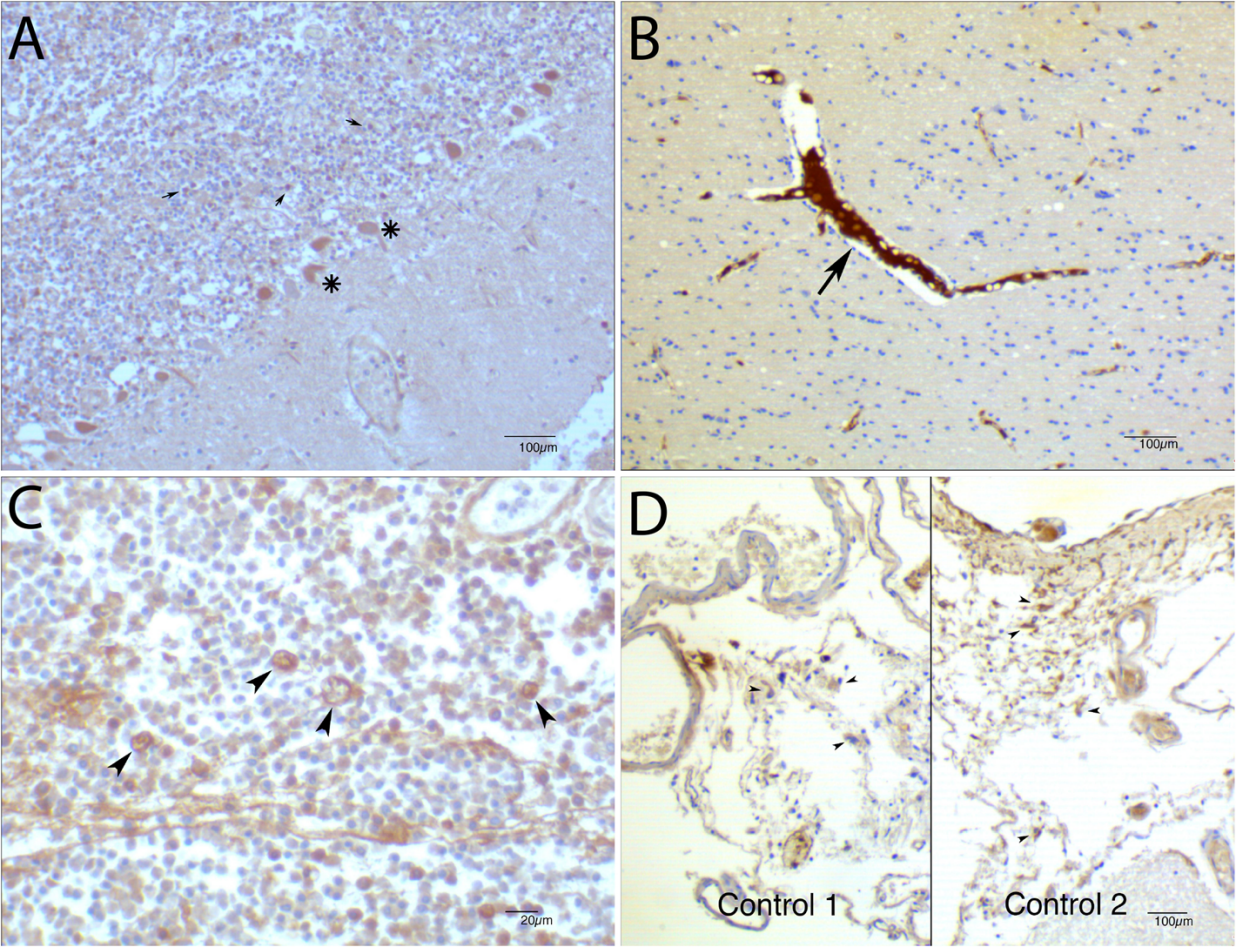
Immunohistochemical staining of brain tissue of a pneumococcal meningitis patient. In the pneumococcal meningitis FH was expressed in the brain parenchymal cells (**a**), with strong expression in Purkinje cells (**a**, asterisk) and in cells in the granular layer (**a**, arrows). In the control case the parenchyma was negative (**b**) and FH expression was confined in the blood within blood vessels (**b**, arrow). Inflammatory cells in the meninges of the pneumococcal meningitis case showed strong expression of FH (**c**) and macrophages show stronger positivity than granulocytes (**c**, arrowheads). In the control case, meningeal cells showed variable intensity FH expression (**d**, arrowheads)

Subsequently, we performed immunohistochemical staining using an anti-human FH antibody to study FH presence in the brain of a pneumococcal meningitis patient and negative control, who died of myocardial infarction. The pneumococcal meningitis case showed FH in the brain parenchymal cells (Fig. 2a), with strong positivity in Purkinje cells and in cells in the granular layer. In the control case, the parenchyma was negative (Fig. 2b) and FH was confined to the lumen of blood vessels. Inflammatory cells in the meninges of the pneumococcal meningitis case showed strong positivity of FH (Fig. 2c) and macrophages show stronger positivity than granulocytes. In the control case, meningeal cells showed variable intensity FH expression (Fig. 2d)

### FH is expressed in a mouse model of pneumococcal meningitis

**Fig. 3:**
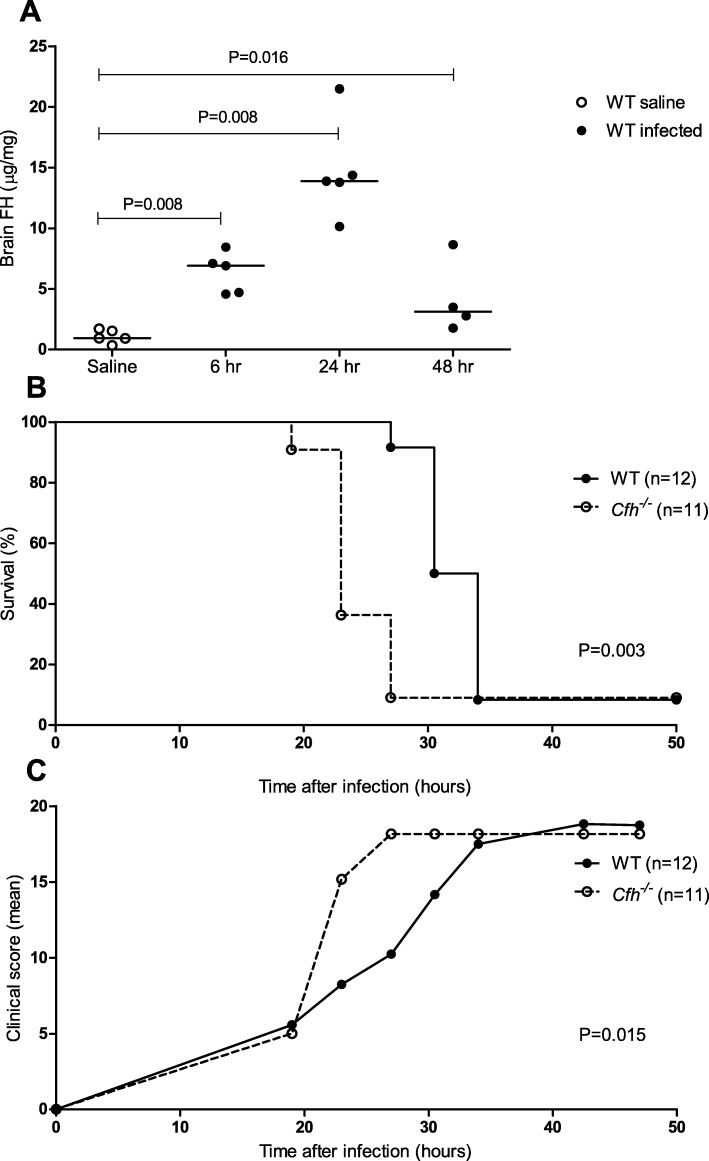
Expression of FH during experimental pneuomococcal meningitis and the effect of FH deficiency on clinical severity and survival. FH brain levels are increased during experimental pneumococcal meningitis at 6, 24, and 48 h after infection compared to saline inoculated mice (**a**). Lines represent median values, *P* values were determined with the Mann-Whitney *U* test. Kaplan-Meier curve of wt and *Cfh*^*−/−*^ mice with pneumococcal meningitis. *P* value was determined with the log-rank test (**b**). Clinical severity scores for *Cfh*^*−/−*^ mice increased faster as compared to wt mice (0.112 vs. 0.088 points/h). *P* value was determined using exponential regression (**c**)

To determine the role of FH during pneumococcal meningitis we used our well-validated pneumococcal mouse model [41]. To verify FH levels in the brain during pneumococcal meningitis 15 wt mice were injected in the cisterna magna with *S. pneumoniae* serotype 3 and euthanized at 6, 24, and 48 h after infection. Mice injected with sterile saline were used as control. One mouse reached an endpoint before the 48 h time point and was taken out of the experiment. Brain FH levels were determined by ELISA and were significantly higher in mice with pneumococcal meningitis at 6 (median 6.92 μg/mg tissue, *P* = 0.008), 24 (median 13.89 μg/mg tissue, *P* = 0.008) and 48 h (median 3.13 μg/mg tissue, *P* = 0.016) after infection compared to saline-inoculated mice (median 0.93 μg/mg tissue, Fig. 3a).

### FH deficiency increases disease severity through secondary C3 depletion in a pneumococcal meningitis mouse model

To explore the role of FH on disease severity during pneumococcal meningitis we compared wt mice with *Cfh*^*−/−*^ mice during a 50-h survival study (*n* = 12 per group). One *Cfh*^*−/−*^ mouse had a limb paresis after intracisternal injection and was taken out of the experiment. All remaining 23 mice showed signs of infection and the first mouse reached an endpoint at 19 h after infection. Overall mortality during the 50 h observation period was 10 of 11 (91%) in the *Cfh*^*−/−*^ mice and 11 of 12 (92%) in the wt mice. *Cfh*^*−/−*^ mice had a significantly shorter survival time compared to wt mice (median survival 23 vs. 32 h, log-rank *P* = 0.003; Fig. 3b). Clinical severity scores increased faster in *Cfh*^*−/−*^ mice compared to wt mice (12% vs. 9% increase in points per hour, exponential regression *P* = 0.015; Fig. 3c).

**Fig. 4.**
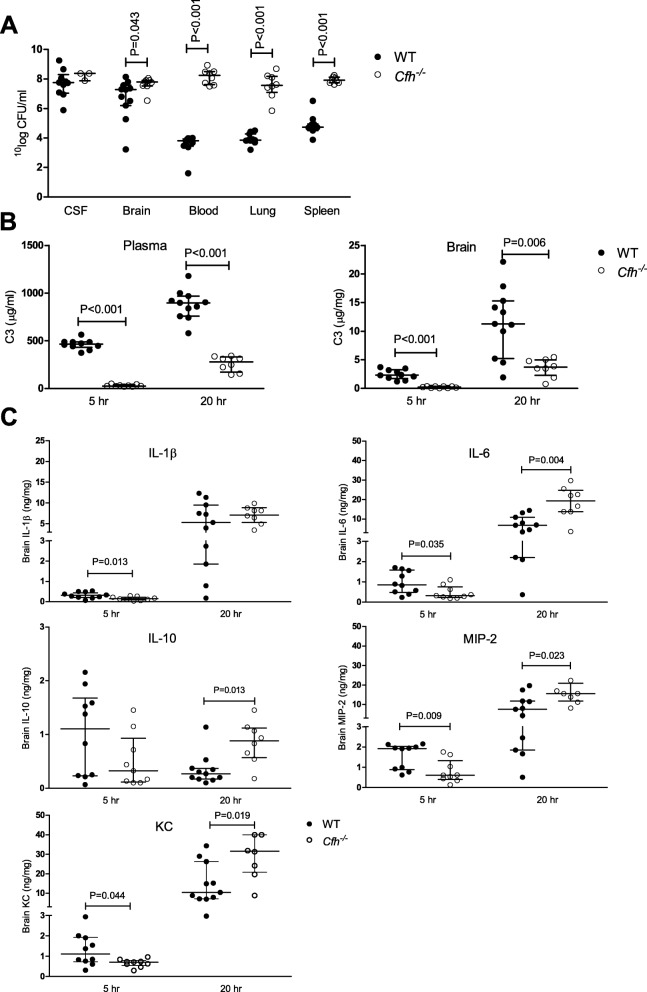
Effect of FH deficiency on bacterial outgrowth, complement, and inflammatory response during experimental pneumococcal meningitis. Bacterial outgrowth in CSF, brain, blood, lung, and spleen of *Cfh*^*−/−*^ and wt mice at 20 h after infection (**a**). Plasma and brain C3 levels were decreased in *Cfh*^*−/−*^ mice compared to wt mice at 5 and 20 h after infection (**b**). *Cfh*^*−/−*^ mice had significantly lower cytokine and chemokine brain levels at 5 h after infection and significant higher cytokine and chemokine brain levels at 20 h after infection (**c**). Data are given as medians and interquartile ranges, *P* values were determined with the Mann-Whitney *U* test

Subsequently, we introduced pneumococcal meningitis in *Cfh*^*−/−*^ and wt mice and euthanized them at 5 (*n* = 10 per group) and 20 h (*n* = 11 per group) after infection. Two *Cfh*^*−/−*^ mice died before the start of the experiment (one per time point) and two *Cfh*^*−/−*^ mice reached an endpoint before the 20-h time point, leaving 17 *Cfh*^*−/−*^mice (5-htime point *n* = 9 and 20-h time point *n* = 8) and 21 wt mice (5-h time point *n* = 10 and 20-h time point *n* = 11). At the 5-h time point bacterial outgrowth was increased in lung tissue in *Cfh*^*−/−*^ mice compared to wt mice (5.60 × 10^4^ vs. 7.50 × 10^3^ CFU/mg tissue, *P* = 0.019). Bacterial outgrowth was increased in *Cfh*^*−/−*^ mice compared to wt mice in blood (1.79 × 10^8^ vs. 6.45 × 10^3^ CFU/ml, *P* < 0.001), brain (median 6.20 × 10^8^ vs. 1.90 × 10^8^ CFU/mg tissue, *P* = 0.043), spleen (8.25 × 10^8^ vs. 5.50 × 10^5^ CFU/mg tissue, *P* < 0.001) and lung (3.73 10^8^ vs. 7.10 10^4^ CFU/mg tissue, *P* < 0.001) at 20 h after infection (Fig. 4a).

FH deficiency has been associated with secondary depletion of circulating C3 due to uncontrolled alternative pathway activation [42]. In our pneumococcal meningitis mouse model *Cfh*^*−/−*^ mice had significantly lower C3 levels in plasma and brain at 5 (26.35 vs. 466 μg/ml plasma, *P* < 0.001 and 0.22 vs. 2.33 μg/mg brain tissue, *P* < 0.001) and 20 h (280.30 vs. 898.30 μg/ml plasma, *P* < 0.001 and 3.70 vs. 11.29 μg/mg brain tissue, *P* = 0.006) after infection (Fig. 4b). At 5 h after infection *Cfh*^*−/−*^ mice had significantly lower brain levels of interleukin (IL)-1β (0.13 vs. 0.30 ng/mg tissue, *P* = 0.013), IL-6 (0.32 vs. 0.86 ng/mg tissue, *P* = 0.035), macrophage inflammatory protein 2 (MIP-2) (0.61 vs. 1.93 ng/mg tissue, *P* = 0.009) and keratinocyte chemoattractant (KC) (0.71 vs. 1.11 ng/mg tissue, *P* = 0.044) compared to wt mice. In contrast, at 20 h after infection brain levels of IL-6 (19.30 vs. 6.76 ng/mg tissue, *P* = 0.004), IL-10 (0.88 vs. 0.27 ng/mg tissue, *P* = 0.013), MIP-2 (15.56 vs. 7.48 ng/mg tissue, *P* = 0.023) and KC (31.55 vs. 10.43 ng/mg tissue, *P* = 0.019) were increased in *Cfh*^*−/−*^ mice compared to wt mice (Fig. 4c). No differences were observed between *Cfh*^*−/−*^ and wt mice in brain levels of IL-10 at 5 h and IL-1β at 20 h after infection. No differences were observed between *Cfh*^*−/−*^ and wt mice in brain albumin content as indication of blood brain barrier disruption.

### Adjuvant treatment with human FH inhibits complement activation but does not improve outcome in a mouse model of pneumococcal meningitis

**Fig. 5:**
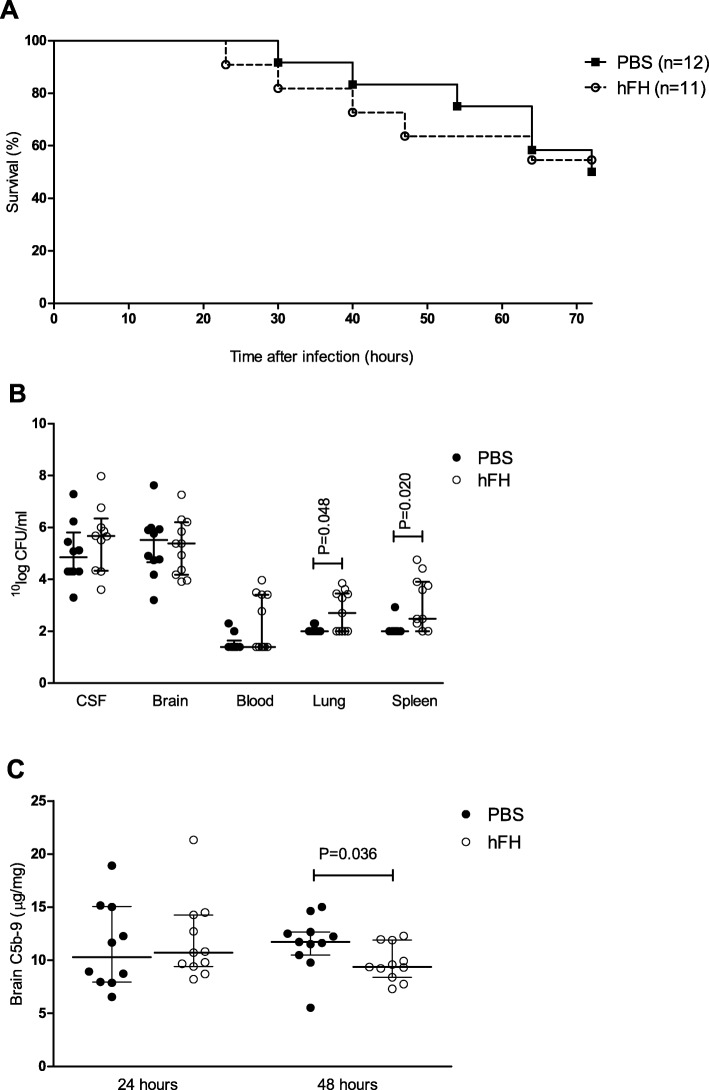
Adjuvant treatment with human FH in experimental pneumococcal meningitis. Mortality rates were similar between hFH- and PBS-treated mice (45% vs. 50%) (**a**). Bacterial outgrowth was increased in hFH-treated mice compared to PBS-treated mice in lung and spleen at 24 h after infection (**b**). Activation of the terminal complement pathway indicated by C5b-9 level was significant lower in mice treated with hFH compared to mice treated with PBS in the brain at 48 h after infection, median 9.37 μg/mg tissue vs. 11.71 μg/mg tissue (**c**). Lines represent median values and error bars are interquartile ranges, *P* values were determined with the Mann-Whitney *U* test

Because our previous experiments showed that FH influences the inflammatory response, we evaluated the effect of adjuvant treatment with purified plasma-derived human FH (hFH) on disease severity in our pneumococcal meningitis mouse model. In a randomized investigator blinded trial wt mice were injected into the cisterna magna with *S. pneumoniae* serotype 3, to mimic the clinical situation mice were treated with intraperitoneal ceftriaxone (100 mg/kg) daily from 16 hours after infection. Mice were randomly assigned to adjuvant treatment with intraperitoneal hFH (1 mg) or PBS at 16 h after infection. In a survival study with 24 mice, one showed a limb paresis after inoculation and was taken out of the experiment. All remaining 23 mice showed clinical signs of infection at 15 h after infection and were randomly assigned to treatment groups (n=11 hFH and n=12 PBS group). The first mouse reached an endpoint at 23 h after infection and the 72-h mortality rates were similar between groups, 5 of 11 (45%) in the hFH group and 6 of 12 (50%) in the PBS group (log-rank *P* = 0.937, Fig. 5a). There was no difference in clinical severity scores.

Subsequently, pneumococcal meningitis was induced in 44 mice for a time-point experiment. One mouse showed a limb paresis after inoculation and was taken out of the experiment. The remaining 43 mice were randomly assigned to the treatment groups: 22 mice received adjuvant treatment with intraperitoneal hFH (11 sacrificed at 24 h and 11 sacrificed at 48 h after infection) and 21 mice with PBS (10 sacrificed at 24 h and 11 sacrificed at 48 h) at 16 h after infection. In all hFH-treated mice, hFH was detectable at 24 and 48 h after infection (8 and 32 h after treatment) in plasma (median 81.20 μg/ml at 24 h and 87.80 μg/ml at 48 h) and brain (median 8.84 μg/mg tissue at 24 h and 10.70 μg/mg tissue at 48 h). Bacterial outgrowth was increased in hFH-treated mice compared to PBS-treated mice in lung (median 5.00 × 10^3^ vs. 1.00 × 10^3^ CFU/mg tissue, *P* = 0.048) and spleen (median 3.00 × 10^3^ vs. 1.00 × 10^3^ CFU/mg tissue, *P* = 0.20) at 24 h after infection (Fig. 5b). There was no difference in bacterial outgrowth in the blood, brain, and CSF at 24 h after infection or in any compartment at 48 h after infection. Activation of the terminal complement pathway indicated by C5b-9 level was significant lower in mice treated with hFH compared to mice treated with PBS in the brain at 48 h after infection (median 9.37 μg/mg tissue vs. 11.71 μg/mg tissue, *P* = 0.036; Fig. 5c). There was no difference in C5b-9 level in plasma at 24 and 48 h after infection. No differences were observed between hFH- and PBS-treated mice in the brain levels of IL-1β, IL-6, MIP, and KC.

Because of our observation that bacterial outgrowth was increased in hFH-treated mice in the systemic compartment at 24 hours with no difference at 48 hours after infection we hypothesized that the pneumococcus benefits from hFH treatment by binding it to evade complement mediated killing before ceftriaxone is effective. We performed a second survival experiment where mice were treated 16 h after inoculation with daily ceftriaxone and at 18 h after inoculation with hFH (1 mg) or PBS (*n* = 12 per group). Two mice showed neurological deficits after inoculation and were excluded from the experiment leaving 11 mice per group. The 72-h mortality rate was similar between hFH- and PBS-treated mice, 7 of 11 (64%) in both (log-rank *P* = 0.897). There was no difference in clinical severity scores.

## Discussion

In our nationwide prospective cohort study of adults with community-acquired bacterial meningitis, we found a functional genetic variant in FH influencing CSF FH level and mortality. The risk (major) allele (G) of the rs6677604 variant is a non-coding SNP located in intron 11 of *CFH* and has previously been described to increase susceptibility for age-related macular degeneration and IgA nephropathy and to decrease susceptibility for systemic lupus erythematosus [46–48]. We now describe this genetic variant to influence an infectious disease. In age-related macular degeneration, the risk major allele (G) was shown to be associated with decreased FH plasma concentration, which is similar to our findings that this allele decreased FH CSF concentrations and is associated with increased mortality in bacterial meningitis [47]. We hypothesize that low-baseline FH levels, associated with the major allele (G) rs6677604, are detrimental due to the lack of inhibition of the complement system during bacterial meningitis resulting in more inflammation and complement mediated damage.

FH concentration was increased during bacterial meningitis in patients and mice with pneumococcal meningitis. The finding that the association between high CSF FH levels and unfavorable outcome was no longer statistically significant after correction for CSF total protein implying that high CSF FH levels are indicative of blood-brain barrier disruption. Indeed, in our human autopsy material, FH was present in brain parenchymal cells in pneumococcal meningitis whereas in our control FH was present only inside the lumen of blood vessels. In mice, FH deficiency increased disease severity through previously described C3 depletion caused by spontaneous activation of C3 due to the absence of inhibition by FH [42]. FH deficiency was associated with an early decrease and late increase of brain cytokine and chemokine levels. The lack of complement activation leads to a decreased initial inflammatory response and a decreased bacterial clearance. The increased bacterial outgrowth causes an increased inflammatory response at the late time point. The smaller difference in bacterial outgrowth between *Cfh*^*−*^*/*^*−*^ and wt mice in the central nervous system compared to the systemic compartment can be explained by the relative immune deficiency in the central nervous system. Complement components are expressed at low levels in the central nervous system. During the course of infection, complement factors are expressed in the central nervous system and can pass the disrupted blood-brain barrier leading to further complement activation and bacterial clearance. This is in agreement with previous findings in experimental pneumococcal meningitis in rabbits depleted of C3 by administering cobra venom with higher bacterial titres in the CSF and C3 deficient mice with increased mortality due to increased bacteremia and systemic complications [18, 19].

The potential anti-inflammatory effect of FH in pneumococcal meningitis triggered us to evaluate the effect of adjuvant hFH in experimental pneumococcal meningitis. Human FH has been shown to enter the central nervous system and inhibit mouse alternative pathway activity in vivo with restored plasma C3 levels in *Cfh*^*−/−*^ mice for at least 48 h after a single intraperitoneal injection [38, 43]. In a mouse model of autoimmune encephalomyelitis, hFH treatment was associated with decreased disease severity, inflammation and demyelination [38]. In our pneumococcal meningitis mouse model, adjuvant treatment with 1 mg hFH at 16 h after infection did not influence disease severity. This is concordant with previous work where hFH treatment did not influence the outcome in a pneumococcal sepsis mouse model [49].

In our treatment model, hFH was detected in plasma and brain of hFH-treated mice. This led to inhibition of complement activation, as indicated by decreased brain levels of C5b-9 in hFH-treated mice at 48 h after infection. However, it did not result in a decreased inflammatory response and decreased disease severity. In hFH-treated mice bacterial outgrowth was increased at 24 h in the systemic compartment. We hypothesize the bacteria benefit from the hFH treatment by binding it to evade complement activation before antibiotic treatment is effective. As described in an in vitro study with human serum serotype 3 pneumococci express, the factor H-binding inhibitor of complement (Hic) to avoid complement attack and opsonophagocytosis [50]. This is in line with previous findings that treatment with hFH decreased bacterial clearance from blood due to reduced pneumococcal C3 opsonization in a murine sepsis model [37]. A second explanation could be that the effect of hFH treatment was limited by the timing of administration, 16 and 18 h after infection, when complement is already activated. Administration of hFH might be beneficial when given earlier during the disease course, but this has no clinical relevance for pneumococcal meningitis patients. Interference of the alternative pathway by targeting other complement components can still be a promising target in pneumococcal meningitis.

Our study has several limitations. First, DNA was not available for all patients and those for whom no DNA was available had a worse outcome compared to those with DNA available. This led to selection bias with a relatively good population for the genetic analysis, which reduced our power to detect an association with unfavorable outcome and death. Second, knockout and wild-type mice were not backcrossed because of time and cost considerations. Therefore we cannot rule out that an unidentified small variation between strains may contribute to the phenotype. However, this does not influence the expression and treatment studies. Third, only the left cerebral hemisphere was used to determine bacterial outgrowth in mouse brain without showing even distribution of the bacteria over both hemispheres. Since the infection is introduced in the CSF in the middle of the cisterna magna we believe the bacteria will spread evenly to the subarachnoid space. Fourth, differences between pneumococcal serotypes may affect the efficacy since strains vary in capability to bind hFH [51–53]. We chose serotype 3 for our pneumococcal meningitis mouse model as it was the most common serotype in our nationwide prospective cohort of community-acquired bacterial meningitis [3]. Although the proportion of serotype 3 cases decreased due to the introduction of conjugate vaccines, it is still among the most common clinical serotypes in pneumococcal meningitis [1, 54]. Additionally *S. pneumoniae* is known to bind human FH but not to murine FH [55]. Ideally, experiments would be performed with humanized FH transgenic mice, but these were not available at the time.

## Conclusions

In conclusion, we show FH has an important role in the pathophysiology of bacterial meningitis and genetic variation influences severity of the disease. Our mouse model shows that treatment with hFH inhibits complement activation during pneumococcal meningitis but did not influence outcome due to detrimental and beneficial effects.

## Supplementary information

**Additional file 1: Table S1.** Baseline characteristics of patients with community-acquired bacterial meningitis with and without DNA available (664 of the 1009 bacterial meningitis episodes (66%). a) Data are number/number evaluated (%) or median (interquartile range). b) Immunocompromise was defined by the use of immunosuppressive drugs, a history of splenectomy, or the presence of diabetes mellitus, alcoholism, as well as patients infected with the human immunodeficiency virus (HIV). c) Score on the Glasgow Coma Scale Score was evaluated in 1008 patients. d) CSF white blood cell count was determined in 639 patients, CSF proteins levels in 634 patients and the CSF/blood glucose ratio in 630 patients with DNA available. CSF white blood cell count was determined in 332 patients, CSF proteins levels in 329 patients and the CSF/blood glucose ratio in 316 patients without DNA available.

## Data Availability

Data of the MeninGene study is available for all researchers at www.MeninGene.eu.

## Abbreviations

C: Complement component
CSF: Cerebrospinal fluid
FH: Complement factor H
GOS: Glasgow Outcome Scale
hFH: Human FH
IL: Interleukin
KC: Keratinocyte chemoattractant
MIP-2: Macrophage inflammatory protein 2
PBS: Phosphate buffered saline
SNP: Single nucleotide polymorphisms
wt: Wild-type
