# Supplementary material for: Complement factor H contributes to mortality in humans and mice with bacterial meningitis
Source: J Neuroinflammation. 2019 Dec 28;16:279. doi: 10.1186/s12974-019-1675-1 (PMC6935240; doi:10.1186/s12974-019-1675-1)
Supplement: Supplementary file 1 — Additional file 1: Table S1. Baseline characteristics of patients with community-acquired bacterial meningitis with and without DNA available (664 of the 1009 bacterial meningitis episodes (66%). a) Data are number/number evaluated (%) or median (interquartile range). b) Immunocompromise was defined by the use of immunosuppressive drugs, a history of splenectomy, or the presence of diabetes mellitus, alcoholism, as well as patients infected with the human immunodeficiency virus (HIV). c) Score on the Glasgow Coma Scale Score was evaluated in 1008 patients. d) CSF white blood cell count was determined in 639 patients, CSF proteins levels in 634 patients and the CSF/blood glucose ratio in 630 patients with DNA available. CSF white blood cell count was determined in 332 patients, CSF proteins levels in 329 patients and the CSF/blood glucose ratio in 316 patients without DNA available. [file 12974_2019_1675_MOESM1_ESM.doc]

**Additional file 1: Table S1. Baseline characteristics of patients with community-acquired bacterial meningitis with and without DNA available (664 of the 1009 bacterial meningitis episodes (66%).** a)

| Clinical characteristics | DNA available (n=664) | No DNA available (n=345) | P value |
| --- | --- | --- | --- |
| Age (years) | 59 (43-68) | 63 (51-73) | P<0.001 |
| Male | 325 (49%) | 184 (53%) | 0.186 |
| Predisposing conditions | 391/664 (59%) | 189/345 (55%) | 0.211 |
| Otitis or sinusitis | 240/663 (36%) | 89/344 (26%) | 0.001 |
| Pneumonia | 54/649 (8%) | 36/327 (11%) | 0.026 |
| Immunocompromised state b) | 162/664 (24%) | 96/345 (28%) | 0.236 |
| Symptoms and signs at admission |  |  |  |
| Symptoms < 24 hr | 229/649 (35%) | 153/331(46%) | 0.281 |
| Headache | 518/609 (85%) | 224/284 (79%) | P<0.001 |
| Neck stiffness | 499/641 (78%) | 223/315(71%) | P<0.001 |
| Temp > 38 °C | 507/663 (76%) | 250/339(74%) | 0.342 |
| Score on Glasgow coma scale c) | 11 (9-14) | 11 (9-14) |  |
| <8 indicating coma | 78/664 (12%) | 57/344 (17%) | 0.033 |
| CSF values d) |  |  |  |
| White blood cell count (/µl) | 4608 (1120-11400) | 2323 (351-7819) | <0.001 |
| Protein (g/l) | 3.7 (3.7-6.0) | 4.0 (2.5-6.0) | 0.099 |
| CSF/blood glucose ratio | 0.05 (0.00-0.28) | 0.02 (0.00-0.17) | <0.001 |
| Causative pathogen |  |  |  |
| S. pneumoniae | 474 (71%) | 248 (72%) | 0.868 |
| N. meningitidis | 78 (12%) | 27 (8%) | 0.053 |
| Other | 103 (16%) | 68 (20%) | 0.092 |
| Score on Glasgow outcome scale |  |  |  |
| 1 – Death | 45 (7%) | 137(40%) | <0.001 |
| 2 – Vegetative state | 1 (0.2%) | 0 | 1.000 |
| 3 – Severe disability | 29 (4%) | 19 (6%) | 0.650 |
| 4 – Moderate disability | 111 (17%) | 49 (14%) | 0.299 |
| 5 – Good recovery | 478 (72%) | 140 (41%) | <0.001 |

a) Data are number/number evaluated (%) or median (interquartile range).

b) Immunocompromise was defined by the use of immunosuppressive drugs, a history of splenectomy, or the presence of diabetes mellitus, alcoholism, as well as patients infected with the human immunodeficiency virus (HIV).

c) Score on the Glasgow Coma Scale Score was evaluated in 1008 patients.

d) CSF white blood cell count was determined in 639 patients, CSF proteins levels in 634 patients and the CSF/blood glucose ratio in 630 patients with DNA available. CSF white blood cell count was determined in 332 patients, CSF proteins levels in 329 patients and the CSF/blood glucose ratio in 316 patients without DNA available.
